# Single-cell transcriptome profiling reveals vascular endothelial cell heterogeneity in human skin

**DOI:** 10.7150/thno.54917

**Published:** 2021-04-19

**Authors:** Qingyang Li, Zhenlai Zhu, Lei Wang, Yiting Lin, Hui Fang, Jie Lei, Tianyu Cao, Gang Wang, Erle Dang

**Affiliations:** Department of Dermatology, Xijing Hospital, Fourth Military Medical University, Xi'an, Shaanxi 710032, P. R. China.

**Keywords:** vascular endothelial cells, single-cell RNA sequencing, skin vessel heterogeneity, skin arteriovenous markers, metabolic diversity

## Abstract

Vascular endothelial cells (ECs) are increasingly recognized as active players in intercellular crosstalk more than passive linings of a conduit for nutrition delivery. Yet, their functional roles and heterogeneity in skin remain uncharacterized. We have used single-cell RNA sequencing (scRNA-seq) as a profiling strategy to investigate the tissue-specific features and intra-tissue heterogeneity in dermal ECs at single-cell level.

**Methods:** Skin tissues collected from 10 donors were subjected to scRNA-seq. Human dermal EC atlas of over 23,000 single-cell transcriptomes was obtained and further analyzed. Arteriovenous markers discovered in scRNA-seq were validated in human skin samples via immunofluorescence. To illustrate tissue-specific characteristics of dermal ECs, ECs from other human tissues were extracted from previously reported data and compared with our transcriptomic data.

**Results:** In comparison with ECs from other human tissues, dermal ECs possess unique characteristics in metabolism, cytokine signaling, chemotaxis, and cell adhesions. Within dermal ECs, 5 major subtypes were identified, which varied in molecular signatures and biological activities. Metabolic transcriptome analysis revealed a preference for oxidative phosphorylation in arteriole ECs when compared to capillary and venule ECs. Capillary ECs abundantly expressed HLA-II molecules, suggesting its immune-surveillance role. Post-capillary venule ECs, with high levels of adhesion molecules, were equipped with the capacity in immune cell arrest, adhesion, and infiltration.

**Conclusion:** Our study provides a comprehensive characterization of EC features and heterogeneity in human dermis and sets the stage for future research in identifying disease-specific alterations of dermal ECs in various dermatoses.

## Introduction

Vascular endothelial cells (ECs), the interior lining of blood vessels, have been recognized as more than inert bystanders but also active participants in intercellular crosstalk. Through regulating metabolic pathways, responding to various cytokines, and recruiting immune cells, ECs have largely participated in multiple diseases such as diabetes, atherosclerosis and cancer 1. Recent single-cell atlas of murine ECs has revealed EC heterogeneity among multiple tissues, including brain, muscle, heart, kidney and liver 2-4, presumably to meet distinct physiological requirements of each tissue 5. For instance, brain ECs link tightly to restrict paracellular diffusion and regulate neuronal migration, whereas liver ECs facilitate a rapid exchange of solutes and modulate liver generation and fibrosis 6,7. In skin tissue, physiological angiogenesis is indispensable for wound healing and hair cycle 8, yet little was known about the specific features of skin ECs at tissue-level.

Blood vessels in skin, the largest and outermost organ confronting various external stimuli, stand at the front line of immune responses. As the gatekeeper locking out circulating immune cells and pro-inflammatory cytokines, cutaneous ECs may alternatively initiate and amplify various inflammatory dermatoses. Recently, we have demonstrated that dermal ECs actively participate in psoriasis development via regulating neutrophil infiltration 9,10. Interestingly, the prominent vessel dilation in psoriatic lesions is mostly seen in papillary dermis but not in reticular dermis, indicating the inhomogeneous EC populations across the vascular bed.

The cutaneous vasculature is elaborately organized throughout the dermis. Small arteries form the deep plexus, from which individual vessels rise to the border between subcutaneous adipose tissue (SAT) and dermis. The deep plexus rises to ascending arterioles to generate the superficial plexus, forming capillary loops that enter the papillary dermis. These capillaries were drained by venules, which form the intermediate plexuses. These vessels are varied in diameter, wall width and basement membrane structure 11. Arterial ECs possess tighter junctions, whereas venous ECs have anticoagulant and procoagulant properties 12. ECs also exhibit diverse metabolic features within the arteriovenous axis. Histopathology reveals diverse susceptible vascular sites in different dermatoses, with a preference of arterioles in antiphospholipid syndrome, capillaries in livedoid vasculopathy, and post-capillary venules in leukocytoclastic vasculitis 13. However, a detailed description of dermal EC subtypes has not been demonstrated.

Here, we investigated the tissue-specific traits and internal heterogeneity of human dermal ECs by single-cell RNA sequencing (scRNA-seq). Dermal ECs were equipped with unique features in transcriptional modulation and gene expressions, when compared to ECs from other tissues. Heterogeneity among dermal EC subtypes, including diverse metabolic preference and immune functions, was discovered. Our study reveals a comprehensive single-cell atlas of human dermal ECs and supports future research as a baseline to identify alterations of dermal EC subpopulations in various skin diseases.

## Results

### Dermal ECs consist of five transcriptionally distinct subtypes

**Figure 1:**
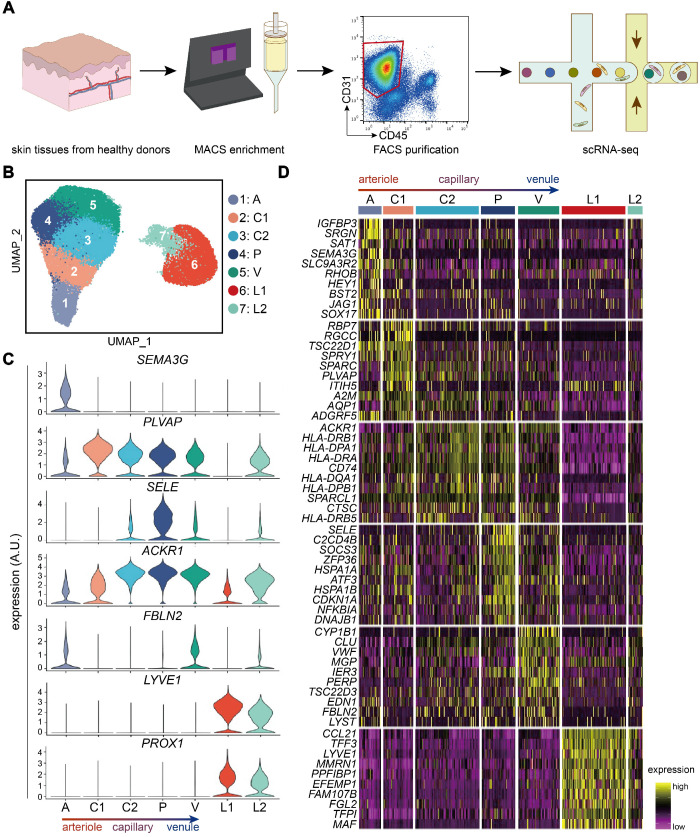
** Transcriptionally distinct subtypes in human dermal ECs via scRNA-seq.** (**A**) Workflow. Skin tissues from 10 healthy donors were used for scRNA-seq. CD31^+^ cell enriched by MACS were further purified to select live CD31^+^ CD45^-^ cells by FACS. 10X Genomics Chromium scRNA-seq was used to profile the cells. (**B**) UMAP plot of 33,265 human dermal endothelial cells, colored by cluster. (**C**) Expressions of *SEMA3G*, *PLVAP*, *SELE*, *ACKR1*, *FBLN2*, *LYVE1*, and* PROX1* in each cluster. y axis represents log-normalized expression. (**D**) Heatmap of single-cell expressions of the top-10 DEGs in each cluster. Cluster A: arteriole ECs; Cluster C1, C2: capillary ECs; Cluster P: post-capillary ECs; Cluster V: venule ECs; Cluster L1, L2: lymphatic endothelial cells. A.U.: arbitrary unit; DEGs: differentially expressed genes; ECs: vascular endothelial cells; FACS: fluorescence activated cell sorting; MACS: magnetic-activated cell sorting; scRNA-seq: single-cell RNA sequencing; UMAP: uniform manifold approximation and projection.

To characterize the heterogeneity of ECs in skin tissue, we established a single-cell atlas of dermal ECs. Normal human skin tissues were collected from 10 healthy donors. Live single CD31^+^ CD45^-^ cells were isolated from digested skin tissues by magnetic-activated cell sorting (MACS) and fluorescence-activated cell sorting (FACS) to enrich endothelial cells, including both vascular and lymphatic endothelial cells (LECs) 14,15 (Figure 1A; Supplementary Figure S1A). These cells were subjected to scRNA-seq by a 10X Genomics-based protocol in 4 separate batches. After rigorous quality control (Supplementary Figure S1B-F), batch-effect correction (Supplementary Figure S1G-H), we excluded 69 contaminating smooth muscle cells (*ACTA2*, *RGS5*), fibroblasts (*COL1A1*), pericytes (*PDGFRA*, *PDGFRB*), red blood cells (*HBA1*, *HBA2*, *HBB*), and immune cells (*PTPRC*) (Supplementary Figure S2). 33,265 high-quality endothelial cells were clustered into 7 subtypes (A, C1, C2, P, V, L1, and L2) by unsupervised clustering (Figure 1B).

Cell annotations were guided by the expression of canonical markers and molecules with inhomogeneous expression patterns (Figure 1C). *SEMA3G*, recognized as an arteriole marker, was exclusively expressed in cluster A 16. Cluster C1, C2, P, and V highly expressed *PLVAP*, which encodes endothelial fenestrae, an ultrastructure more often seen in capillaries 11. Moreover, *SELE*, important for neutrophil rolling in post-capillary venules, was highly expressed in cluster P 17. Cells in cluster C2, P, and V were enriched with *ACKR1*, which was reported to be highly expressed in post-capillary venules and venules but not arterioles 18. Both cluster A and V exhibited high levels of *FBLN2*, critical in collagen-containing extracellular matrix and elastic intima, which are less seen in capillaries 11. *LYVE1* and* PROX1*, markers of LECs, were highly expressed in L1 and L2 15,19. Taken together, cluster A was primarily recognized as arteriole ECs, C1-2 as capillary ECs, P as post-capillary venule ECs, V as venule ECs, and L1-2 as LECs.

Each dermal EC subtype had more than 50 differentially expressed genes (DEGs) (Supplementary File 1). Heatmap analysis of the top-10-ranking DEGs revealed distinct signatures of dermal EC subtypes and LECs (Figure 1D). Interestingly, clusters in adjoining position along the arteriovenous axis shared similar gene expression signatures. For instance, several genes such as *TSC22D1*, *A2M*, and *ADGRF5* were expressed in both cluster A and C1, suggesting the arteriole-like identity of cluster C1. Cluster C2 and P shared expressions of human lymphocyte antigen class II (HLA-II) molecules, although cluster P selectively expressed *SELE* and *HSPA1A*. The expression of shared DEGs by ECs from more than one subtype indicated that these subtypes were phenotypically related to each other. Because the primary goal of this study was to illustrate the features of dermal ECs, LECs were only briefly characterized (Figure 1D; Supplementary File 1), and ECs were subsampled from our scRNA-seq dataset for further analysis.

### Dermal ECs exhibit zonational and phenotypic heterogeneity

**Figure 2:**
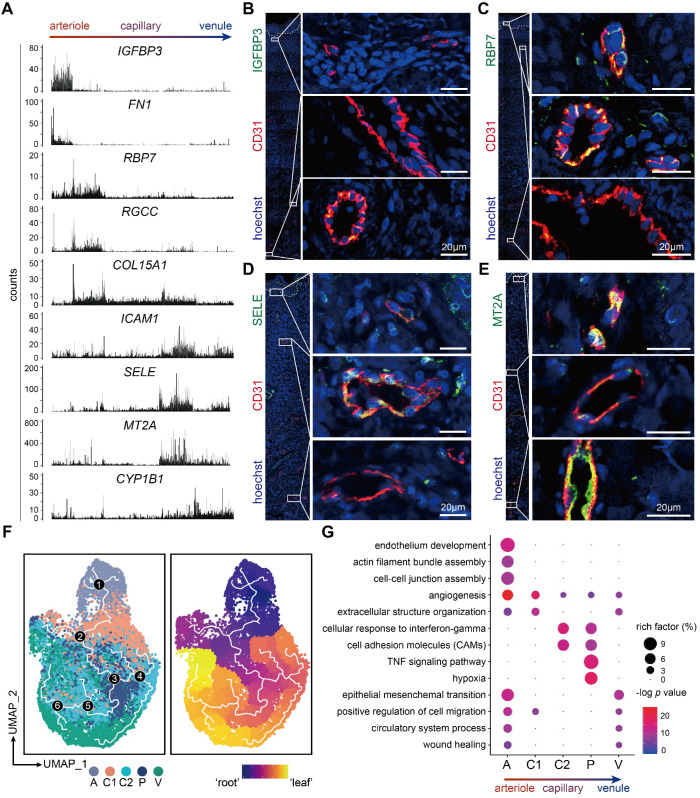
** Heterogeneous expressions and functions of dermal ECs along the arteriovenous axis.** (**A**) Zonational expression of representative genes across dermal vascular bed. ECs are rearranged in an arteriovenous order. y axis represents counts. (**B-E**) Immunofluorescence of IGFBP3, RBP7, SELE, MT2A and CD31. Images of CD31^+^ ECs in the superficial, intermediate, and deep plexus are zoomed in. The image is representative of 11 biological replicates (limbs, n = 4; trunk, n = 4; foreskin, n = 3). The white dotted line marks the interface between epidermis and dermis. Scale bars represent 20 µm. (**F**) Single-cell trajectory of dermal EC subtypes by pseudotime analysis. The white line traces the trajectory. ECs in the left panel are colored by clusters, and white numbers in black circles mark the branch nodes of the pseudotime trajectory. ECs in the right panel plot are colored by pseudotime. Color scale: yellow, 'leaf' of the trajectory; purple, 'root' of the trajectory. (**G**) GO pathway enrichment analysis of the marker genes of dermal EC clusters. The color represents -log *p* value, and the size indicates the rich factor. Cluster A: arteriole ECs; Cluster C1, C2: capillary ECs; Cluster P: post-capillary ECs; Cluster V: venule ECs. ECs: vascular endothelial cells; GO: gene ontology; UMAP: uniform manifold approximation and projection.

To explore the arteriovenous markers in skin tissue, the 5 dermal EC subtypes were reordered into a one-dimensional range, from arteriole, capillary, to venule ECs, which is referred to as the cellular 'zonation' 20. Along this zonational range, various genes were found with diverse expression patterns. For instance, *IGFBP3* and* FN1* were selectively enriched in the left end of this range, *RBP7*,* RGCC*, and *COL15A1* enriched in the left-middle part, whereas *ICAM1*, *SELE, MT2A*, and *CYP1B1* enriched in the right end (Figure 2A). To further confirm their potential roles as arteriovenous markers, we examined the tissue expressions of representative genes via immunofluorescence. Of note, consistent with the zonational expression, IGFBP3 was positively stained in ECs of the deep plexus of human skin, which is mostly arterioles and venules (Figure 2B). RBP7 was co-stained with CD31 in the superficial and intermediate plexus of human skin (Figure 2C). Meanwhile, cell adhesion molecules such as ICAM1 and SELE (also known as CD62E), which peaked in the right end of the zonation, were expressed in the intermediate plexus, which are mostly composed of venules, but not in the superficial or deep plexus (Figure 2D and Figure 6D). Besides, MT2A showed higher expressions in the superficial and deep plexus of human skin (Figure 2E). The heterogeneous expressions of IGFBP3, RBP7, SELE, and MT2A along the vascular bed were further validated in murine femoral arteries and veins (Supplementary Figure S3).

Considering that the dermal ECs are in different phenotypic states, we examined the developmental trajectory via peudotime analysis to explore cell differentiation and activation. The phenotypic states of dermal ECs occurred on a tightly organized trajectory, starting from cluster A and ending with cluster V (Figure 2F). This result further confirmed our arteriovenous zonation mentioned above. Interestingly, cells in C2 and P, are rather mingled with each other in the trajectory, suggesting a developmentally indistinguishable phenotype shared among them. This could be reasonably perceived since these sections of the vessel hold similar biological functions such as cell adhesion and intravasation. To further illustrate their functional divergence, gene ontology (GO) analysis of marker genes in each EC subtype were explored (Figure 2G). Cluster A was involved in angiogenesis and cell junction assembly, indicating a relatively tight and compact intercellular structure in arterioles. Cluster C2 and P were more active in cellular response to interferon-gamma and cell adhesion, while cluster P selectively enriched cytokine signaling and hypoxia pathways. Therefore, these findings denote a heterogeneous transcriptional profile among dermal ECs, which gives rise to the heterogeneous functions of cutaneous vasculature in homeostasis and diseased states.

### Dermal ECs are distinguished from ECs in other tissues by tissue-specific traits

**Figure 3:**
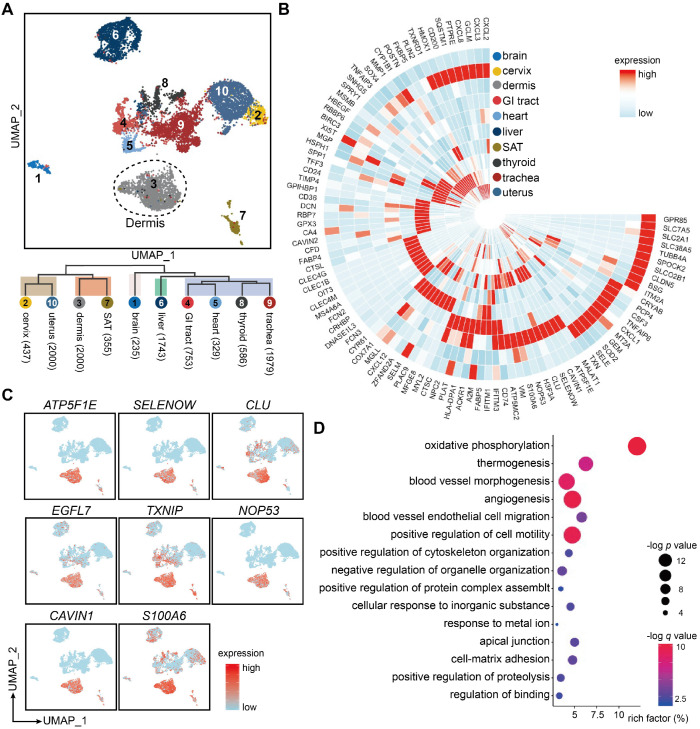
** Tissue-specific heterogeneity of ECs from 10 different tissues.** (**A**) UMAP visualization of 10,417 ECs from 10 different tissues/organs with cells colored by their origin. The numbers in brackets after each tissue/organ name indicate the cell numbers in the corresponding tissue/organ. The dotted line circles ECs originated from dermis. (**B**) Circular heatmap of average expressions of top-10 DEGs in ECs from 10 tissues/organs. (**C**) Signature gene expressions of dermal ECs. (**D**) GO annotation enrichment analysis of DEGs in dermal ECs. x axis represents the rich factor, the circle size represents the -log *p* value, and the color represents the -log *q* value. DEGs: differentially expressed genes; ECs: vascular endothelial cells; GI: gastrointestinal; GO: gene ontology; SAT: subcutaneous adipose tissue; UMAP: uniform manifold approximation and projection.

Recent studies have indicated organ-specific signatures in ECs from different organs in mice and human fetuses 2,21, however few studies have focused on ECs from skin tissue. To depict the tissue-specific traits of human dermal ECs, we analyzed scRNA-seq data of 10,417 ECs from 10 different tissues/organs, including brain, cervix, gastrointestinal (GI) tract, heart, liver, thyroid, trachea, uterus (GSE134355, 22), SAT (GSM3717979, 23), and dermis (our data). Uniform manifold approximation and projection (UMAP) visualization revealed prominent transcriptomic heterogeneity among ECs from different tissues/organs, with cells congregated mainly by their tissue/organ origin (Figure 3A). ECs from dermis and SAT or uterus and cervix, which are anatomically and functionally adjacent, were located in a relatively close distance in the UMAP plot. GO analysis further corroborated that similar functions were shared in ECs from dermis and SAT, including the regulation of collagen metabolic process and monocyte differentiation (Supplementary Figure S4A). These results suggest that ECs from adjacent tissues share a similar expression profile.

Then, we explored the EC marker genes for each tissue/organ (Supplementary File 2). Heatmap of the top-10-ranking marker genes revealed diverse signatures of ECs from different tissues/organs, except for the cervix and uterus, which expressed overlapping marker genes (Figure 3B). Of note, some of these EC marker genes were selectively expressed in a single tissue/organ (such as *SLC7A5*, *MYL2*, *FCN2* in brain, heart, and liver, respectively), whereas other EC marker genes (such as *COX7A1*,* MGP*, and *XIST*) were conserved across two or more tissues/organs. Brain ECs were characterized by high expressions of the members in solute carrier family, whereas ECs in skin and GI tract were distinguished by high levels of immune-related genes such as *CD74*, *HLA-DRA*, *HLA-DPA1*, and *ACKR1*.

Because the primary goal of this study was to focus on the skin tissue, we further investigated the tissue-specific features of ECs from dermis (Figure 3C). Genes such as *ATP5F1E*, *SELENOW*, *CLU* and *TXNIP*, which are related to mitochondria and redox signaling 24, were selectively expressed in dermal ECs. *EGFL7*, *NOP53*, and *S100A6*, modulating angiogenesis and cell proliferation 25, were also enriched in ECs from dermis and SAT, which may be related to the upregulated transcription factors of the JUN family (Supplementary Figure S4B). *CAVIN1*, a key component of caveolae, was highly expressed in ECs from both dermis and SAT, indicating a relatively active process in endothelial vesicular trafficking and signal transduction in skin tissue 26. GO analysis revealed that dermal ECs participated in oxidative phosphorylation, angiogenesis, EC migration and pathways related to cytoskeleton organization. Besides, dermal EC were highly active in biological processes in responses to metal ion, inorganic substance, and apical junction and cell-matrix adhesion (Figure 3D). These results point out the transcriptional uniqueness of dermal ECs at tissue-level.

### Dermal ECs possess inter- and intra-tissue metabolic heterogeneity

**Figure 4:**
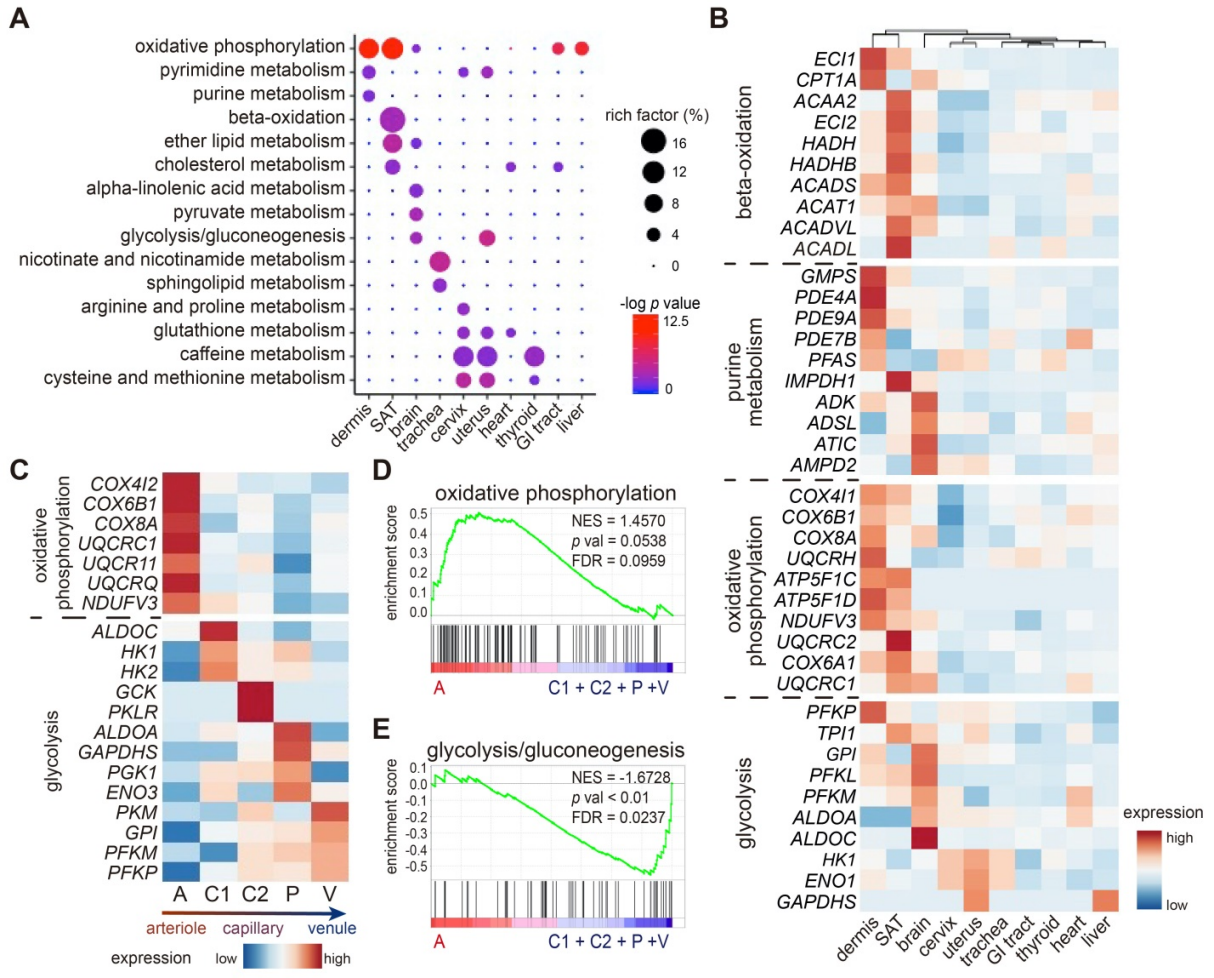
** Metabolic heterogeneity in ECs across tissues and within dermis.** (**A**) KEGG analysis of metabolic pathways across ECs from different tissues/organs. The circle size represents the rich factor, and the color represents the -log *p* value. (**B**) Heatmap of expressions of representative metabolic genes among ECs from different tissues. (**C**) Heatmap of expressions of oxidative phosphorylation and glycolytic genes across 5 dermal EC clusters. (**D**) GSEA analysis of the dataset of dermal ECs against the oxidative phosphorylation geneset. (**E**) GSEA analysis of the dataset of dermal ECs against the glycolysis/gluconeogenesis geneset. A positive enrichment score on the y axis indicates positive correlation with cluster A and a negative enrichment score indicates a negative correlation. Cluster A: arteriole ECs; Cluster C1, C2: capillary ECs; Cluster P: post-capillary ECs; Cluster V: venule ECs. ECs: vascular endothelial cells; FDR: false discovery rate; GI: gastrointestinal; GSEA: gene set enrichment analysis; KEGG: kyoto encyclopedia of genes and genomes; NES: normalized enrichment score; SAT: subcutaneous adipose tissue.

EC metabolism is closely correlated to endothelial functions. To explore the metabolic heterogeneity across ECs from different tissues/organs, kyoto encyclopedia of genes and genomes (KEGG) analysis was performed (Figure 4A). Oxidative phosphorylation pathway was highly enriched in ECs from dermis, SAT, liver, and GI tract, whereas glycolysis/gluconeogenesis pathway was more active in ECs from brain and uterus. ECs from both SAT and brain enriched lipid metabolism, with β-oxidation and cholesterol metabolism higher in SAT ECs, and alpha-linolenic acid metabolism higher in brain ECs. Moreover, nicotinate and nicotinamide metabolism was selectively enriched in trachea ECs, which may indicate higher risks of smoke exposure.

The three major substrates for energy and biomass production in ECs are mainly confined to fatty acids, amino acids, and glucose. We therefore compared the inter-tissue expressions of metabolic genes within the fatty acid β-oxidation, purine, oxidative phosphorylation, and glycolysis pathways (Figure 4B). Consistent with the KEGG analysis (Figure 4A), ECs from SAT highly expressed genes related to fatty acid β-oxidation, such as *ACAA2*, *ECI2*, *ACADVL*, etc. ECs from both dermis and brain held high expressions of purine metabolism-related genes, but the specific pathways varied. Dermal ECs enriched genes within the 3',5'-cyclic AMP degradation and GMP metabolism such as *PDE4A*, *PDE7B*, *GMPS*, and *PFAS*, whereas brain ECs enriched IMP biogenesis molecules such as *ADSL*, *ATIC*, and *AMPD2*. As for glucose metabolism, ECs from skin and brain expressed higher enzymatic glucose genes than ECs from other tissues/organs. *UQCRH*, *ATP5F1D*, and *NDUFV3*, key genes in oxidative phosphorylation, were highly expressed in ECs from dermis and SAT. However, glycolytic genes such as *GPI*, *PKFL*, and *ALDOC* were more enriched in ECs from the brain, which relies on glucose as the sole fuel 27.

We further questioned whether this metabolic preference towards oxidative phosphorylation was consistent across subtypes in skin ECs. A heatmap of representative metabolic genes revealed a gradual switch from oxidative phosphorylation to glycolysis in dermal EC subtypes along the blood flow (Figure 4C). GSEA results reconfirmed that arteriole ECs were more active in oxidative phosphorylation, other than glycolysis and gluconeogenesis pathway, indicating a distinct preference of energy production along the blood flow (Figure 4D-E). To sum up, these results strongly suggest a heterogeneous metabolic profile in ECs from different tissues/organs and from different subpopulations within dermis.

### Dermal capillary ECs are actively involved in immune responses

**Figure 5:**
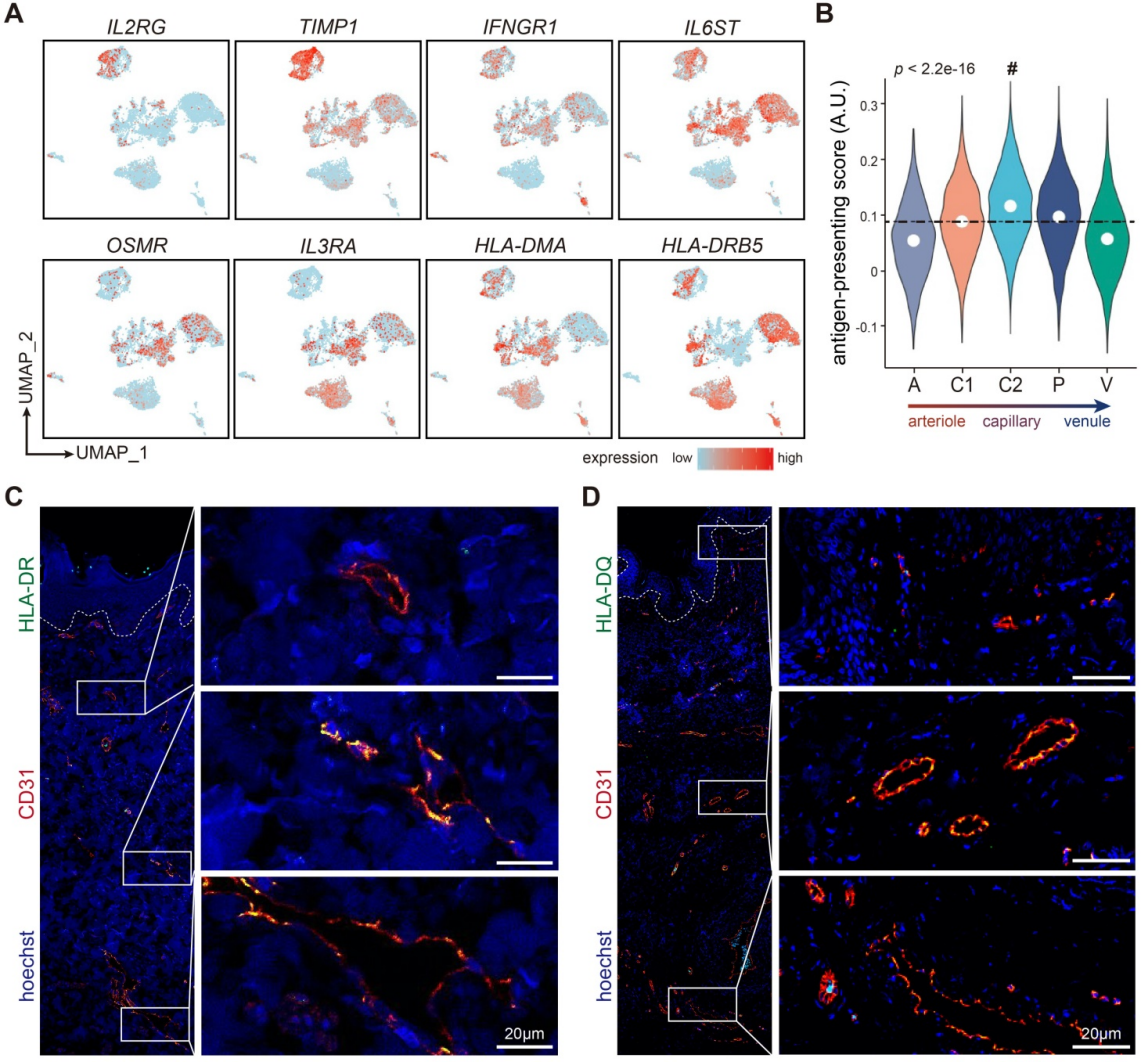
** The expressions of HLA-II genes in dermal capillary ECs.** (**A**) Expressions of selected cytokines and receptors, and HLA-II molecules in ECs from 10 different tissues/organs. (**B**) Antigen-presenting scores of cells in 5 dermal EC subtypes. The mean score is labeled with a dotted line. Cluster C2 with the highest score is indicated by a hash key. (**C, D**) Immunofluorescence of HLA-DR, HLA-DQ, and CD31. Images of CD31^+^ ECs in superficial, intermediate, and deep plexus are zoomed in. The image is representative of 11 biological replicates (limbs, n = 4; trunk, n = 4; foreskin, n = 3). The white dotted line marks the interface between epidermis and dermis. Scale bars represent 20 µm. Cluster A: arteriole ECs; Cluster C1, C2: capillary ECs; Cluster P: post-capillary ECs; Cluster V: venule ECs. A.U.: arbitrary unit; ECs: vascular endothelial cells; HLA-II: human lymphocyte antigen class II; UMAP: uniform manifold approximation and projection.

As ECs stand at the first line confronting circulatory cells and substrates, they play essential immunomodulatory roles in various diseases 28,29. Interestingly, ECs from different tissues/organs possess diverse immune features (Supplementary File 3). For instance, *IL2RG* and *TIMP1* was highly expressed in liver ECs, whereas *IFNGR1* was highly expressed in SAT ECs. *IL6ST* and *OSMR* were enriched in ECs from uterus, cervix, trachea and GI tract, while *IL3RA* was highly expressed in ECs from trachea, dermis, and SAT. Of note, ECs from both skin and GI tract shared high expressions of HLA-II molecules such as *HLA-DMA* and *HLA-DRB5* (Figure 5A).

As non-professional antigen presenting cells, ECs, equipped with HLA-II molecules, are able to initiate immune responses. However, Figure 1D showed that HLA-II genes such as *HLA-DRB1* and* HLA-DPA1* were exclusively expressed in cluster C2 and P, suggesting that the capability to present antigen and activate immune cells was inhomogeneous among different dermal EC subtypes. By measuring the antigen-presenting scores of each dermal EC clusters (Supplementary Table S1), we observed that cluster C2 (capillary ECs) scored the highest, indicating its enhanced antigen presenting function via HLA-II molecules (Figure 5B). To further investigate their functional diversity, immunofluorescence of human skin verified positive expression of HLA-DR and HLA-DQ with CD31 in the intermediate plexus, but not in the superficial and deep plexus, which are mostly consisted of arterioles, post-capillary venules, and venules (Figure 5C-D). The results above denote that dermal capillary ECs play a critical role in immune surveillance and CD4^+^ T cell activation via enhanced expression of HLA-II molecules.

### Dermal post-capillary venule ECs are susceptible cellular adhesion site

**Figure 6:**
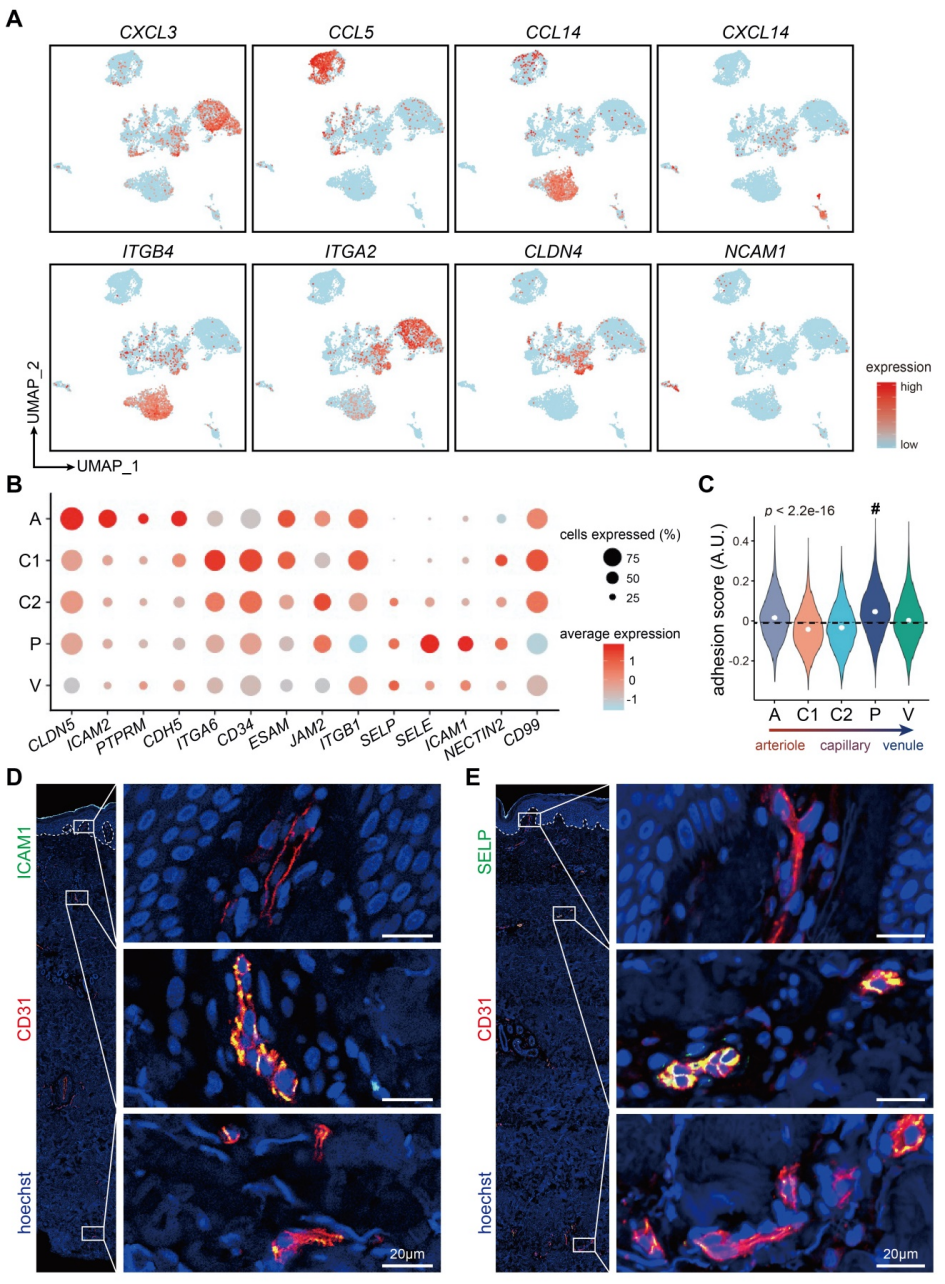
** Susceptible cellular adhesion site in dermal post-capillary venules.** (**A**) Gene expressions of representative chemokines and receptors, integrins, and adhesion molecules in ECs from 10 different tissues/organs. (**B**) Dot plot for expressions of adhesion molecules in dermal EC subtypes. The color represents scaled average expression of adhesion molecules in each subtype, and the size indicates the proportion of cells expressing adhesion molecules. (**C**) Adhesion scores of cells in 5 dermal EC subtypes. The mean score is labeled with a dotted line. Cluster P with the highest score is indicated by a hash key. (**D, E**) Representative immunofluorescence images of ICAM1, SELP, and CD31 in human skin. Images of CD31^+^ ECs in superficial, intermediate, and deep plexus are zoomed in. The image is representative of 11 biological replicates (limbs, n = 4; trunk, n = 4; foreskin, n = 3). The white dotted line marks the interface between epidermis and dermis. Scale bars represent 20 µm. Cluster A: arteriole ECs; Cluster C1, C2: capillary ECs; Cluster P: post-capillary ECs; Cluster V: venule ECs. A.U.: arbitrary unit; ECs: vascular endothelial cells; UMAP: uniform manifold approximation and projection.

Recent studies have updated our understanding towards the role of ECs, which are more than a passive lining of the vessels, but also an active recruiter to initiate inflammatory responses in local sites in inflammatory diseases 30. We therefore examined the expressions of chemokines and receptors, integrins, and cell adhesion molecules across ECs from different tissues/organs (Figure 6A; Supplementary File 3). *CXCL3*, involved in neutrophil-mediated inflammation 31, were highly expressed in cervix and uterus ECs, whereas *CCL5*, a chemoattractant for blood monocytes, memory T helper cells and eosinophils 32, was exclusively expressed in liver ECs. *CCL14* and *CXCL14*, involved in monocyte activation and recruitment 33, were selectively expressed in ECs from dermis and SAT, respectively. Integrins such as *ITGB4* was exclusively expressed in dermal ECs, whereas *ITGA2* was highly expressed in ECs from uterus, cervix, and trachea. ECs from trachea exclusively expressed adhesion molecules such as *CLDN4*, whereas *NCAM1*, involved in neuron adhesion and axonal fasciculation 34, were selectively expressed in brain ECs.

Adhesion molecules were further compared among 5 dermal EC clusters (Figure 6B). *CLDN5*, *PTPRM*, and *CDH5*, critical in intercellular adhesion between ECs, were highly enriched in cluster A, indicating compact cellular junctions in arterioles. Of note, *SELE*, *SELP* (also known as *CD62P*) and *ICAM1*, mediating cellular adhesion between ECs and circulatory cells, were enriched in cluster P. Consistently, cluster P in dermal ECs scored the highest when measuring the adhesion score of dermal EC populations, based on the expression of genes related to neutrophil adhesion to ECs (Figure 6C; Supplementary Table S2). As expected, immunofluorescence of SELE (Figure 2B), SELP, and ICAM1 (Figure 6D-E) in human skin was co-stained with CD31 in the intermediate plexus, but not in the superficial or the deep plexus. Together, these data suggest the heterogeneous functions of circulatory cell recruitment and adhesion in along the vascular bed residing in different tissues and in different layers throughout dermis.

## Discussion

Comparison of transcriptomes of ECs is commonly applied to determine the molecular basis underlying the heterogeneity along the blood flow. In our study, using scRNA-seq and detailed analyses of transcriptional similarity, we depict a detailed single-cell transcriptional atlas of ECs from human skin tissue, which were overlooked before. We show that dermal ECs, unlike ECs derived from other human tissues, own tissue-specific signatures such as *ATP5F1E*, *S100A6*, *SELENOW*, *CAVIN1*, *etc.*, and diverse metabolic and immune features. Moreover, 5 subtypes are identified within dermal ECs and are located sequentially in pseudotime. Via detailed analyses and tissue verification, several marker genes with differentiated expression pattern in cutaneous vasculature are demonstrated such as *IGFBP3*, *RBP7*, *MT2A*, *ICAM1*, *SELE*, and *SELP*. Furthermore, we discovered heterogeneity in metabolism, immune activation, and cell adhesion among EC subtypes from cutaneous vasculature.

Depending on the type of organ in which they reside, ECs hold diverse molecular and functional properties, which lead to inter-organ heterogeneity in the vascular tree 35. Consistent with previous studies in murine or human fetal tissues 2,21, we observed that ECs can be clustered based on their tissue origin, and ECs from anatomically adjacent tissues are similar at transcriptional level, which further indicates the close correlation between ECs and tissue/organ functions. ECs from brain, skin, GI tract, and liver, which are active organs in need of energy to confront neural signals or external stimuli, are metabolically more active than ECs from other tissues/organs. Interestingly, oxidative phosphorylation in ECs from dermis, SAT, liver, and GI tract is more enhanced, whereas glycolysis/gluconeogenesis pathway is more active in ECs from brain. In addition, the distribution of cytokines, chemokines and receptors, HLA-II molecules, and integrins is varied among ECs from different tissues/organs. In cutaneous tissue, ECs are highly expressed with *CXCL14* and* CCL14* but expressed relatively low levels of* CCL5* and *CXCL3*, suggesting that dermal ECs might possess higher potential to recruit and activate monocytes rather than neutrophils. HLA-II genes such as *HLA-DMA* and *HLA-DRB5* are highly expressed in skin ECs as compared to ECs from brain, trachea, and thyroid, which could explain the higher incidence rate of dermal vasculitis in acute graft-versus-host disease 36, systemic sclerosis 37, and cutaneous leukocytoclastic vasculitis 38.

The molecular and functional diversity in ECs has been investigated in several organs such as brain, liver and lung 3,4,39, yet the heterogeneous characteristics of dermal ECs remain largely unknown. Within cutaneous tissue, we identified 5 EC subtypes that is cluster A, C1, C2, P, and V, which are sequentially distributed along the pseudotime trajectory indicating the inheritance in development. Although there are markers for ECs in arteries and veins, these markers may not all precisely apply to arterioles and venules in cutaneous microcirculation. For instance, NR2F2, reported as a marker gene for veins 40, was expressed in capillary and venule ECs in our scRNA-seq data, and showed little expressions at protein level (Supplementary Figure S5). Moreover, arteries can be discriminated against veins by the surrounding smooth muscle cells, however, these differential characteristics in cutaneous vasculature are rather blurred under light microscopy, as smooth muscle cells and pericytes exist in both cutaneous arterioles and venules 41. To try to fill in the blanks, we discovered 4 potential arteriovenous marker genes via zonation analysis and immunofluorescence validation. Based on our data, IGFBP3 and RBP7 were identified as arteriole markers, while SELE and MT2A as venule markers in dermal vasculature. Therefore, our findings may be applied to the diagnosis and identification of various skin vascular diseases and the in-depth study in differential functions of dermal EC subtypes in the future.

EC metabolism is closely connected to their cellular functions such as angiogenesis 42, barrier integrity 43, inflammation 44, and cellular crosstalk 45. Proof-of-principle studies have demonstrated the diverse metabolic traits in EC subtypes. In a vessel sprout, for instance, tip and stalk ECs feature glycolysis and fatty acid oxidation, respectively 46. However, the metabolic traits in EC subtypes from the cutaneous vasculature remain largely unknown. Via analyzing metabolic transcriptomes, we observed a switch from oxidative phosphorylation to glycolysis along the cutaneous arteriovenous axis, which is in accordance with previous findings in pulmonary microvascular ECs 47. The enhanced oxidative phosphorylation in arteriole ECs may be due to higher oxygen pressure 48, shear stress 49, Notch signaling 50, and signaling interactions with smooth muscle cells 51. The preference for glycolysis in capillary and post-capillary venule dermal ECs not only primes them for proliferation in a hypoxic environment, but also could provide higher total cellular ATP contents when external glucose is non-limiting, which coincides with the proliferative characteristics of the superficial plexus in psoriasis and wound healing 52. Moreover, EC metabolism is tightly related to inflammation and immune system. Given that glucose metabolism impacts on VE-cadherin endocytosis 43 and the glycosylation of endothelial glycocalyx layer 53, EC metabolism can modulate endothelial barrier integrity, and hence adhesion and intravasation of immune and cancer cells. Lowering glycolytic flux down-regulates the expressions of cell adhesion molecules such as ICAM1 via decreasing NF-kB signaling 43. In our data, capillary and post-capillary venule ECs were more active in immune process such as cell adhesion and cytokine signaling. Notably, both higher expressions of glycolytic genes and cell adhesion molecules have been found in cluster P (post-capillary venule ECs). Whether this relatively higher potential in cell adhesion is reflected by an increase in glycolysis in cluster P, and if so whether the metabolism is a key driver or a subsequent bystander require further detailed verifications.

ECs are the essential element throughout immune responses, from presenting chemoattractants on the luminal side of vessels, promoting the rolling, firm arrest, adhesion and crawling of various immune cells, to facilitating immune cell activation through direct contact 54. It has been reported that glomerular ECs could generate high levels of chemokines such as CXCL8 and promote neutrophil chemotaxis in systemic lupus erythematosus 55. Our previous study has demonstrated that cutaneous ECs induce neutrophil recruitment, which leads to a cascade of inflammatory events in psoriasis 10. These findings support the immunomodulatory role of ECs. In current study, we have analyzed the process of immune cell recruitment, adhesion, and activation from the perspective of ECs, and discovered the high expressions of *ICAM1*, *SELE* (*CD62E*), and *SELP* (*CD62P*) in cluster P, suggesting a susceptible adhesion site in post-capillary venules. In addition, we discovered that cluster C2 (capillary ECs) expresses high levels of HLA-II molecules, which are sufficient to induce CD4^+^ T cell proliferation and are involved in expansion of regulatory T cells or T helper 17 cells 56. These data indicate that dermal ECs, especially in capillaries and post-capillary venules, are actively involved in inflammatory responses and immune activation. Therefore, our study provides additional support in investigating the cutaneous innate immune responses and uncovering the pathogenesis of inflammatory and autoimmune skin diseases.

We have to admit some limitations exist. Skin samples used for scRNA-seq in our study were collected from limbs, trunk, and foreskin. We acknowledge that ECs from different locations exhibit several expressional divergences, yet the magnitude of which is limited, as only 3 locations were included (Supplementary Figure S6). Further detailed studies of dermal ECs from more locations such as scalp, hands, and feet are required. In addition, ECs from male donors had the majority in our data, leading to a biased gender contribution. To investigate tissue-specific features of dermal ECs, we performed meta-analysis of our data and previously reported data, which provided an unprecedented human single-cell atlas 22. However, we cannot exclude the bias derived from different sequencing methods used in scRNA-seq of ECs from dermis and SAT (10X Genomics) and ECs from the other 8 tissues (Microwell-seq). As our results mostly rely on data at transcriptional level, further protein analysis and functional experiments are warranted to corroborate the putative role of each EC phenotype.

So far, the heterogeneity of dermal ECs in inflammatory dermatoses has not been addressed. Our study sets the baseline for future research in identifying disease-specific changes in dermal EC subtypes and may help further understand the active roles of ECs in metabolism, vascular remodeling, and immune responses.

## Methods

### Human skin samples

Surgical skin tissue discards used for scRNA-seq and immunofluorescence staining were obtained from 10 and 11 individuals respectively at Xijing Hospital. The detailed clinical information of age, gender, tissue anatomical location, and type of surgery were provided in Supplementary Table S3 (for scRNA-seq) and Supplementary Table S4 (for immunofluorescence staining). The dissected skin tissues were free of skin lesions assessed by two dermatologists independently. Skin tissues for scRNA-seq were immediately processed for ECs isolation, and skin samples for immunofluorescence were fixed in formalin and embedded in paraffin. The samples were used in full agreement with our institutional guidelines and with the approval of the Ethics Committee of the Xijing Hospital at the Fourth Military Medical University (reference number KY20183019-1). Written consents from donors were obtained.

### Mice

3 female wild-type C57BL/6J mice aged 8 weeks were purchased from the Department of Laboratory Animal Medicine of Fourth Military Medical University. All mice were housed at the experimental animal lab in a specific pathogen free facility with individual ventilated cages. Mice were provided with free access to a regular rodent chow diet. After anesthesia, the femoral artery and vein isolated with fine scalpels were fixed in formalin and embedded in paraffin. All experimental protocols were performed in accordance with NIH guidelines and were approved by the Review Committee for the Use of Animals of the Fourth Military Medical University.

### ECs isolation from human dermis

Immediately after surgery, human skin tissues were transferred into RPMI-1640 medium (Hyclone, Logan, UT, USA) on ice. After removing SAT, skin tissues were cut into small pieces using fine scalpels and transferred into RPMI-1640 medium containing 2.5 mg/mL Dispase II (Gibco, Grand Island, NY, USA) overnight (4 °C) to separate the epidermis from the dermis. Once separated, the dermis was cut into homogenate and digested for 40 min in a 38 °C water-bath (265 r/min) with RPMI-1640 medium containing 1 mg/mL collagenase type 4 (Worthington, Freehold, NJ, USA), 0.2 mg/mL hyaluronidase (Sigma-Aldrich, St Louis, MO, USA), 0.2 mg/mL DNase I (Sigma-Aldrich), and 10 mM HEPES (Gibco). The digestion was terminated by diluting the cell suspension with 10 mL pre-cooled washing buffer (4 °C). Single cell suspension was proceeded to select CD31^+^ cells using the CD31 MicroBead Kit (Miltenyi Biotec, Germany) according to the manufacturer's instructions. After washed with washing buffer, the enriched CD31^+^ single cell suspension was stained with mouse anti-human CD31 antibody conjugated with FITC (Biolegend, San Diego, CA, USA) and mouse anti-human CD45 antibody conjugated with APC (Biolegend) for 25 min (4 °C), and 7-AAD for 5 min (4 °C) before FACS using MoFlo XDP cell sorter (Beckman Coulter, Brea, CA, USA). Single cells were gated with the FSC-Height versus SSC-Log Height plot, and dead cells were excluded based on 7-AAD dead cell stain. Live single CD31^+^CD45^-^ cells, recognized as endothelial cells, were sorted into the RPMI-1640 medium (Hyclone). Flow cytometry data were analyzed using FlowJo software (Ashland, OR, USA).

### Library preparation, sequencing and data pre-processing

Cell viability was examined by trypan blue staining (Sigma-Aldrich) and controlled with a threshold set >90%. Freshly sorted endothelial cells were immediately processed for library preparation and sequencing by Genergy Bio-technology (Shanghai) Co. Ltd. according to 10X Genomics guidelines. Each sample was sequenced, with, on average, a cell number of 11937 in each batch (batch 1: 1,807; batch 2: 918; batch 3: 23,894; batch 4: 21,130), a sequencing depth of 22,537 reads per cell (batch 1: 48,666; batch 2: 422,167; batch 3: 13,239; batch 4: 13,455), sequencing saturation rate of 60.8% (batch 1: 75.1%; batch 2: 94.5%; batch 3: 35.3%; batch 4: 38.2%), and 1,373 median genes per cell (batch 1: 1,687; batch 2: 2,690; batch 3: 1,291; batch 4: 1,382). Demultiplexing, read alignment and quality control were performed at Genergy Bio-technology (Shanghai) Co. Ltd. using the 10X Cell Ranger package (*ver* 3.0.1).

### Data processing, integrating and clustering

Gene expression matrixes of 4 samples were further processed in R (*ver* 3.6.3). Data from the 4 batches were integrated via Reciprocal Principal Correlation Analysis using the *Seurat* package (*ver* 3.1.2) 57. For quality control, genes expressed in less than 3 cells, and cells expressed less than 200 genes were first removed. We filtered out low-quality cells by taking the number of counts and features, and the percent of mitochondrial and ribosomal genes into account via the *quickPerCellQC* function (nmads = 1.5) in the *Scater* package (*ver* 1.14.6) 58. Then, fast MNN was performed to correct batch effect across samples in *SeuratWrappers* (*ver* 0.1.0). Clustering among single cells was achieved using the *FindNeighbors* (dims = 1:10) and the *FindClusters* (resolution = 0.4) functions in the *Seurat* package. 69 contaminating cells expressing markers of smooth muscle cells (*ACTA2*, *RGS5*), fibroblasts (*COL1A1*), pericytes (*PDGFRA*, *PDGFRB*), red blood cells (*HBA1*, *HBA2*, *HBB*), and immune cells (*PTPRC*) were discarded before further analysis. Data was normalized and 2,000 variable genes were detected. Variabilities of the number of counts, features and percentage of mitochondrial and ribosomal genes were regressed out using the *ScaleData* function. To compare the distribution and expressional differences across cells from limbs (batch 1), trunk (batch 2), and foreskin (batch 3 and 4), we randomly subsampled our data to equalize the cell numbers of ECs from different anatomical locations. DEGs of ECs from different locations were identified using the *DEsingle* package (*ver* 1.6.0) with a cutoff Benjamini-Hochberg adjusted *p* value of 0.05, a normalized average expression of greater than 0.5, and a normalized fold change of greater than 2. These DEGs were uploaded for GO analysis using Metascape (http://metascape.org).

### Comparison of ECs derived from different tissues/organs

To explore the heterogeneity across ECs from different human tissues/organs, we used the publicly available processed data and metadata. According to clustering cell types labeled by original authors, ECs from brain (cerebellum and temporal lobe), cervix, dermis, GI tract (stomach, duodenum, ileum and transverse colon), heart, liver, thyroid, trachea, uterus (GSE134355 22), and SAT (GSM3717979 23) were extracted and combined with dermal ECs in our data. To avoid tissue-specific differences being overwhelmed by substantial amounts of ECs from dermis and uterus, 2,000 ECs were randomly sampled from scRNA-seq data of dermis and uterus respectively. In total, 10,417 ECs originated from 10 different human tissues/organs were integrated into a single matrix and further analyzed.

### Gene expression analysis

The* FindAllMarkers* function (min.pct = 0.25, logfc.threshold = 0.25) in the *Seurat* package was applied to identify DEGs of dermal endothelial cells from 7 clusters or of ECs from 10 tissues/organs. In dermal endothelial cells, 193, 89, 57, 114, 67, 255, and 25 DEGs were found in cluster A, C1, C2, P, V, L1, and L2, respectively (Supplementary File 1). In ECs from 10 different tissues/organs, 70, 74, 242, 15, 29, 62, 267, 38, 140, and 113 DEGs were identified in the brain, cervix, dermis, GI tract, heart, liver, SAT, thyroid, trachea, and uterus, respectively (Supplementary File 2). These DEGs were selected for GO or KEGG analysis using Metascape (http://metascape.org). Gene set enrichment analysis was achieved using GSEA software (*ver* 4.0.3, UC San Diego and Broad Institute, CA and MA, USA). Pseudotime analysis was carried out using the *Monocle3* package (*ver* 0.2.0) 59. Assuming all ECs are in the same developmental pathway and arteriole ECs as the 'root', a pseudotime trajectory linking 5 dermal EC clusters were drawn via *plot_cells* function. To perform transcription factor network inference, metadata containing ECs from 10 different tissues was subsampled by randomly selecting 235 cells from each tissue and analyzed using the *SCENIC* package (*ver* 1.1.2-2) and the *AUCell* package (*ver* 1.8.0) 60. The antigen-presenting scores and adhesion scores of dermal ECs were calculated based on the expressions of genes related to antigen presentation (Supplementary Table S1) and neutrophil adhesion (Supplementary Table S2) using the *AddModuleScore* function in the *Seurat* package.

### Data visualization

To visualize expressions, violin plots, feature plots and heatmaps were generated by the *VlnPlot*, *FeaturePlot*, *DoHeatmap* functions in the *Seurat* package or the *Pheatmap* package (*ver* 1.0.12, https://CRAN.R-project.org/package=pheatmap), respectively. The *ggplot2* (*ver* 3.2.1, https://ggplot2.tidyverse.org) and the *ggsci* (*ver* 2.9, https://CRAN.R-project.org/package=ggsci) packages were used to draw and edit UMAP plots, dot plots, stacked bar plots. A circular heatmap showing the top-10-ranking DEGs of ECs from different tissues/organs was drawn using the *circlize* package (*ver* 0.4.9) 61.

### Immunofluorescence staining

Formalin-fixed paraffin-embedded human skin samples (n = 11) and murine femoral vessel tissues (n = 3) were sectioned in 4 μm. After deparaffinization and dehydration, antigen retrieval was performed for the sections in Tris-EDTA buffer at 98 °C for 20 min. The sections were blocked with goat normal serum for 1 h and incubated overnight at 4 °C with primary antibodies. The following primary antibodies were used: anti-CD31 (1:400, ab199012, Abcam, UK; 1:1000, 10148-T62, Sino Biological, China; 1:200, 550274, BD Biosciences Pharmingen, San Diego, CA, USA), anti-IGFBP3 (1:100, sc-135947, Santa Cruz Biotechnology, Dallas, TX, USA), anti-RBP7 (1:50, MA5-24514, Invitrogen, Carlsbad, CA, USA), anti-SELE (1:100, A2191, ABclonal, China), anti-MT2A (1:100, A17438, ABclonal), anti-HLA-DQ (1:200, ab23632, Abcam), anti-HLA-DR (1:200, ab136320, Abcam), anti-ICAM1 (1:200, ab109361, Abcam), anti-SELP (1:100, ab178424, Abcam), and anti-NR2F2 (1:200, ab41859, Abcam). After washing three times with PBST, these sections were incubated in dark for 1 h at room temperature with secondary antibodies including goat anti-mouse IgG cy3 (1:200, EK012, Zhuangzhibio, China), goat anti-rabbit IgG Alexa Fluor 488(1:1000, ab150077, Abcam), goat anti-rat IgG Cy5 (1:500, ab6565, Abcam). Images were captured with a confocal microscope (LSM880, Carl Zeiss Microscopy GmbH, Germany) using the same exposure parameters. Negative controls that were stained with secondary antibodies were included in all experiments. Images were analyzed with ZEN software (*ver* 1.1.2.0, Carl Zeiss Microscopy GmbH).

### Statistical analysis

To assess statistical significance for the DEGs in 5 dermal EC subtypes and ECs derived from 10 different tissues/organs, we used Wilcoxon Rank Sum test in R. To analyze significance for adhesion score and antigen-presenting score among 5 dermal EC subtypes, we used One-way ANOVA with post hoc Tukey**'**s test through R package *ggpubr* (*ver* 0.2.4, https://CRAN.R-project.org/package=ggpubr).

## Supplementary Material

Supplementary figures and tables.

Supplementary File 1.

Supplementary File 2.

Supplementary File 3.

## Abbreviations

ECs: vascular endothelial cells
DEGs: differentially expressed genes
GI: gastrointestinal
GO: gene ontology
GSEA: gene set enrichment analysis
HLA-II: human lymphocyte antigen class II
KEGG: kyoto encyclopedia of genes and genomes
LEC: lymphatic endothelial cell
MACS: magnetic-activated cell sorting
FACS: fluorescence-activated cell sorting
SAT: subcutaneous adipose tissue
SCENIC: single-cell regulatory network inference and clustering
scRNA-seq: single-cell RNA sequencing
UMAP: uniform manifold approximation and projection
