# Supplementary material for: Single-cell transcriptome profiling reveals vascular endothelial cell heterogeneity in human skin
Source: Theranostics. 2021 Apr 19;11(13):6461–76. doi: 10.7150/thno.54917 (PMC8120211; doi:10.7150/thno.54917)
Supplement: Supplementary file 1 — Supplementary figures and tables. [file thnov11p6461s1.pdf]

Supplementary figures and legends

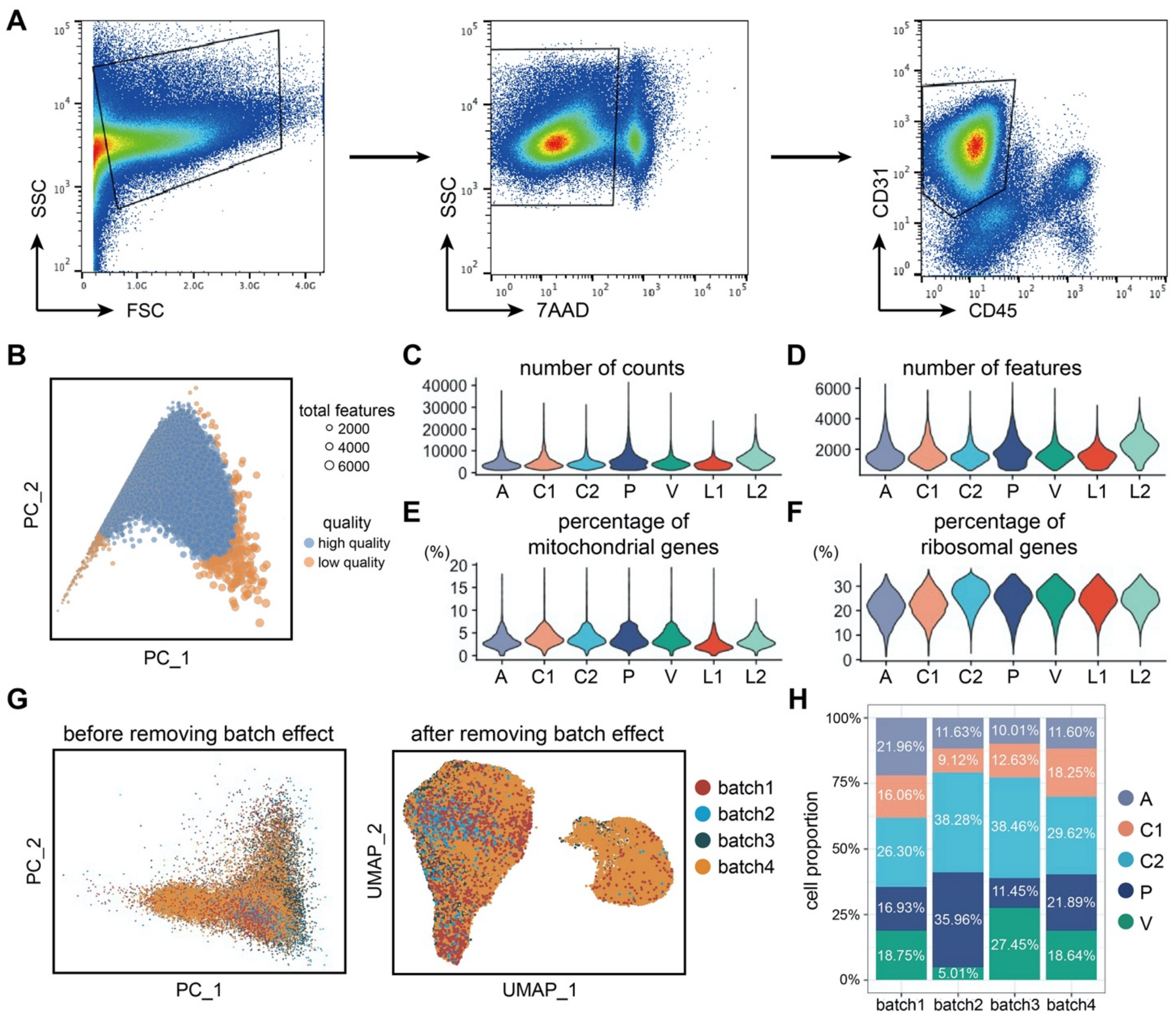

Figure S1. Endothelial cell sorting, quality control, and data integration.

(**A**) Gating logic to sort live CD31^{+} CD45^{-} cells via flow cytometry. (**B**) PCA plot showing high-quality cells (blue) and low-quality cells (orange). Each circle represents a cell. Circle size indicate total features in the cell. (**C-F**) Violin plots showing the numbers of counts and features, percentages of mitochondrial and ribosomal genes after quality control. (**G**) PCA and UMAP plot of cells before and after batch effect removing, respectively. Cells are colored by batch. (**H**) Cell composition of each dermal EC cluster in four batches.

Cluster A: arteriole ECs; Cluster C1, C2: capillary ECs; Cluster P: post-capillary ECs; Cluster V: venule ECs; Cluster L1, L2: lymphatic endothelial cells.

PCA: principal component analysis; UMAP: uniform manifold approximation and projection.

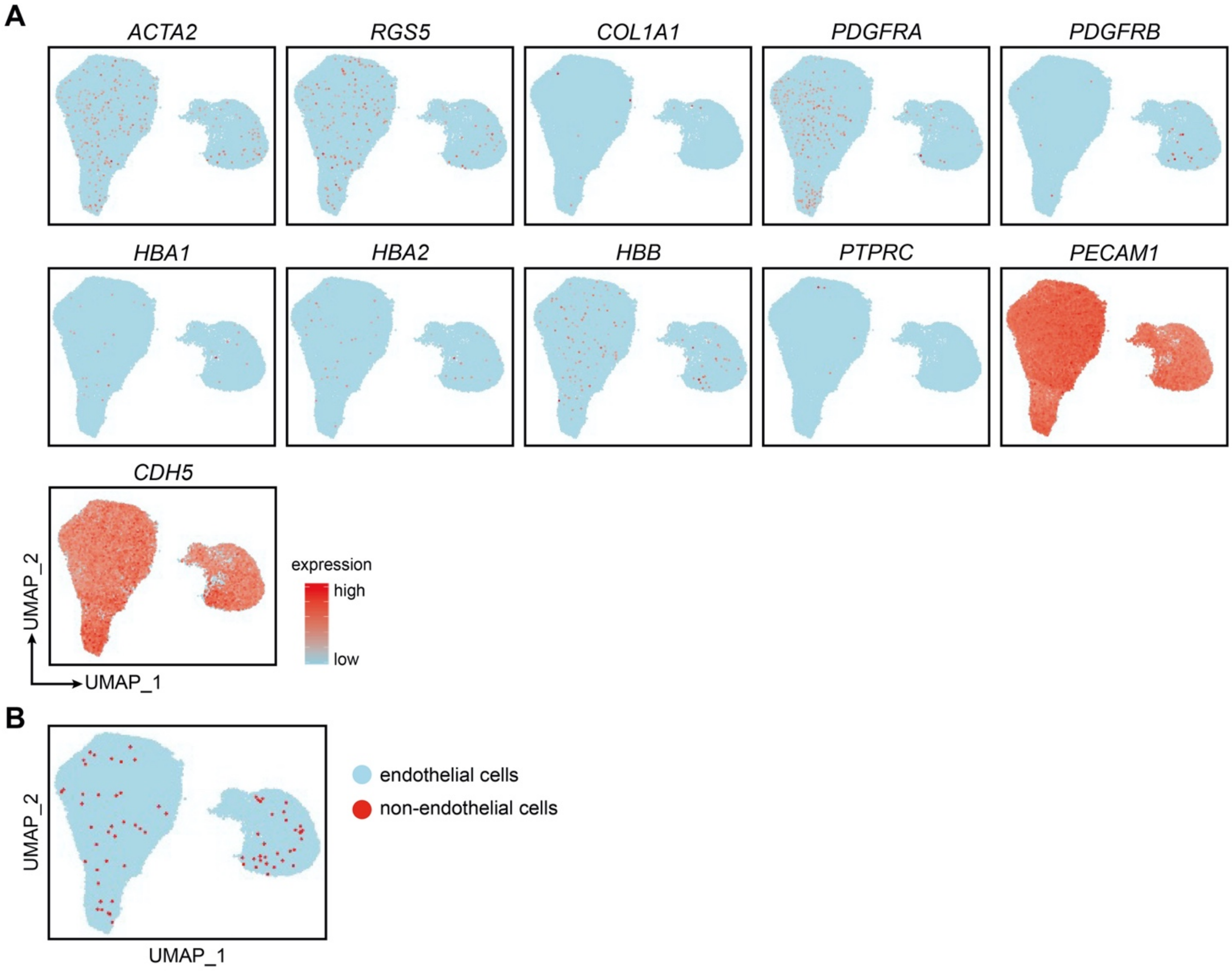

Figure S2. Distinguishing and filtering out non-endothelial cells.

(**A**) Expressions of markers of smooth muscle cells (*ACTA2*, *RGS5*), fibroblasts (*COL1A1*), pericytes (*PDGFRA*, *PDGFRB*), red blood cells (*HBA1*, *HBA2*, *HBB*), immune cells (*PTPRC*), and endothelial cells (*PECAM1*, *CDH5*) in single cell data. (**B**) UMAP plot showing endothelial cells and non-endothelial cells.

UMAP: uniform manifold approximation and projection.

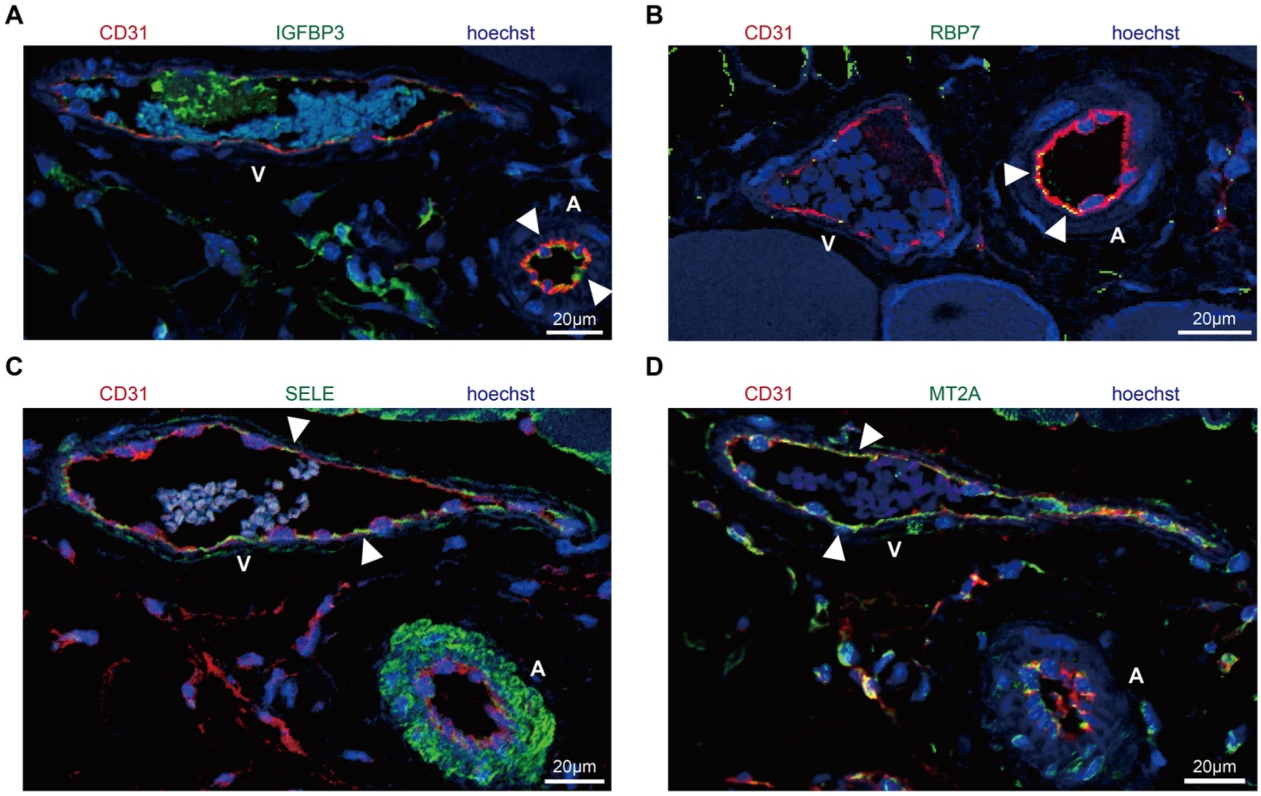

Figure S3. Validation of potential arteriovenous markers in femoral artery and vein.

(**A**-**D**) Immunofluorescence of IGFBP3, RBP7, SELE, MT2A, and CD31 in murine femoral artery and vein. A stands for artery, and V stands for vein. Original magnification 200×; scale bar: 20 μm; n = 3. White arrowheads point out positive co-staining of marker genes and CD31.

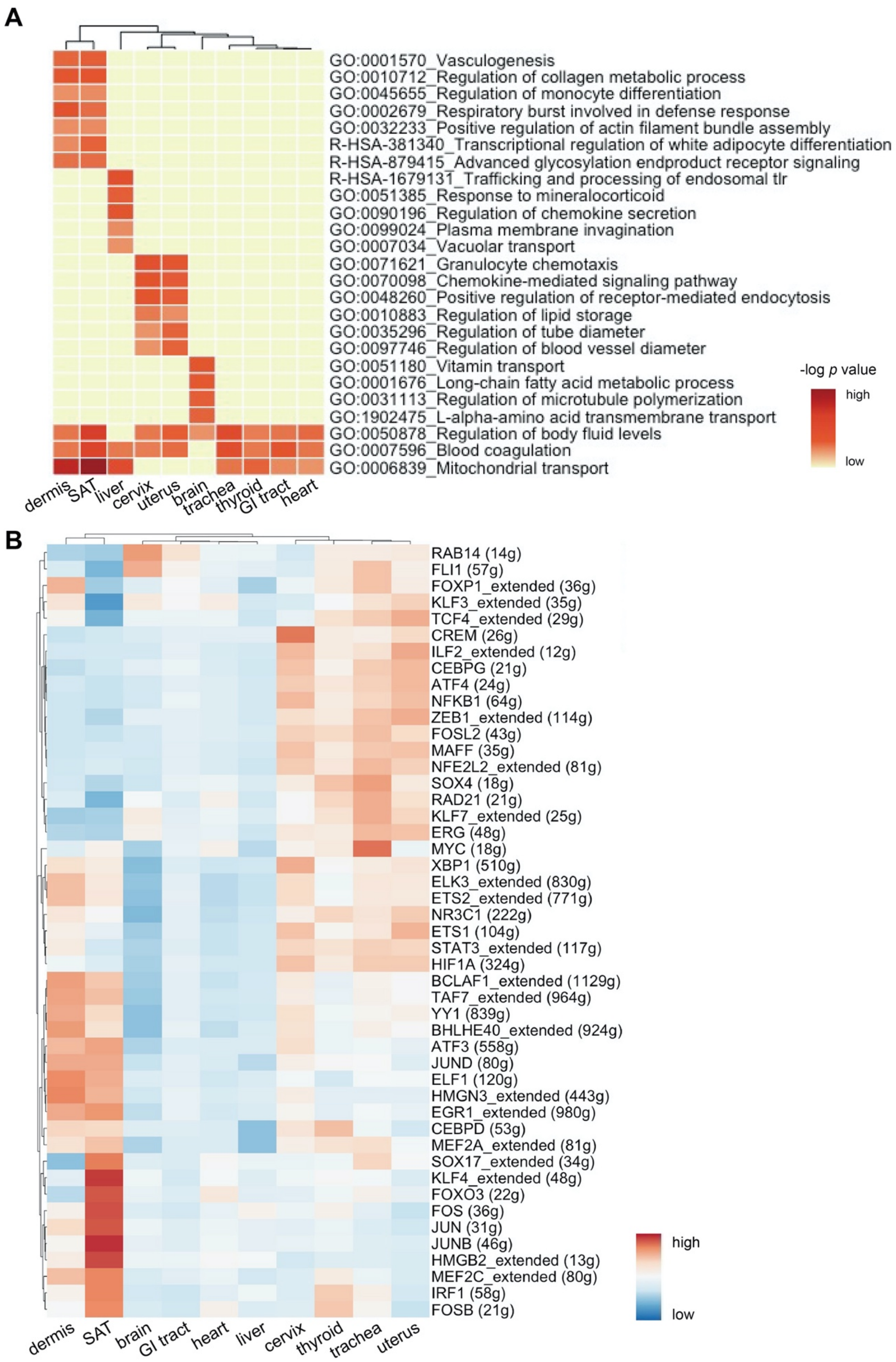

Figure S4. Pathways and transcriptional factors enriched in ECs from 10 different tissues/organs.

(A) Heatmap of the enriched GO pathways in ECs from 10 different tissues/organs. the color represents the -log *p* value. (**B**) Heatmap of the single-cell regulatory network inference and clustering in ECs from different tissues/organs. Numbers in brackets indicate the regulons for transcription factors.

ECs: vascular endothelial cells; GI: gastrointestinal; GO: gene ontology; SAT: subcutaneous adipose tissue.

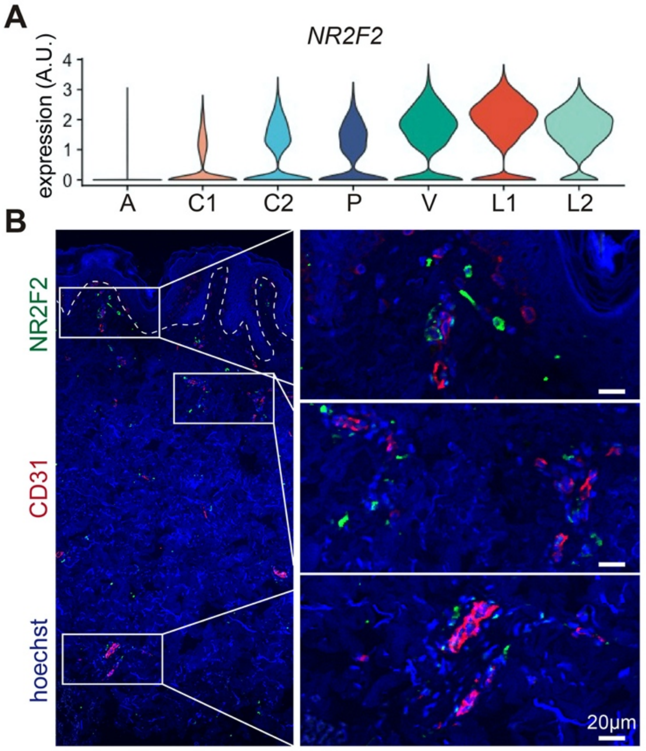

Figure S5. Expressions of *NR2F2* in dermal endothelial cells at mRNA and protein levels.

(**A**) mRNA levels of *NR2F2* in dermal endothelial cell clusters in single-cell RNA sequencing data.

(**B**) Protein levels of NR2F2 in dermal vasculature via tissue immunofluorescence. Images of $CD31^+$ ECs in the superficial, intermediate, and deep plexus are zoomed in. The image is representative of 3 biological replicates. The white dotted line marks the interface between epidermis and dermis. Scale bars represent 20 μm.

Cluster A: arteriole ECs; Cluster C1, C2: capillary ECs; Cluster P: post-capillary ECs; Cluster V: venule ECs.

A.U.: arbitrary unit.

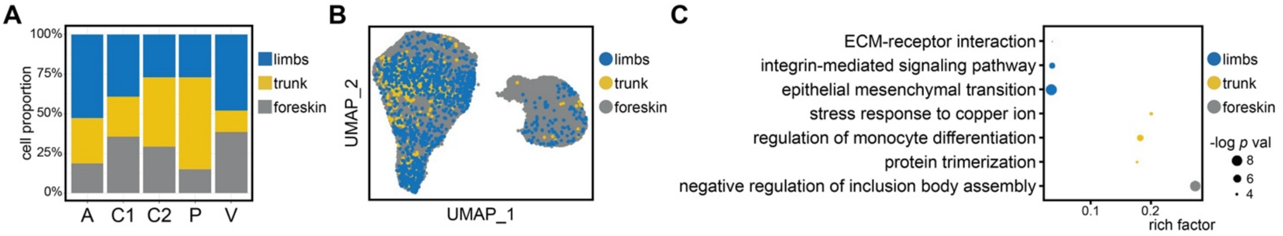

Figure S6. Composition of dermal ECs derived from different anatomical locations.

(**A**) The cell proportions of dermal ECs from different anatomical locations in each dermal EC cluster. (**B**) UMAP plot of dermal endothelial cells colored by anatomical location. (**C**) Pathway analysis of DEGs of dermal ECs derived from limbs, trunk, and foreskin. y axis represents the rich factor. The circle size represents the -log *p* value, and the color represents the anatomical location.

Cluster A: arteriole ECs; Cluster C1, C2: capillary ECs; Cluster P: post-capillary ECs; Cluster V: venule ECs.

DEGs: differentially expressed genes; ECs: vascular endothelial cells; UMAP: uniform manifold approximation and projection.

Supplementary tables

Table S1. Genes for measuring antigen-presenting score.

Genes: GO_ANTIGEN_PROCESSING_AND_PRESENTATION_OF_PEPTIDE_OR_POLYSACCHARIDE_ANTIGEN_VIA_MHC_CLASS_II
AC027237.1
ACTR10
ACTR1A
ACTR1B
AP1B1
AP1G1
AP1M1
AP1M2
AP1S1
AP1S2
AP1S3
AP2A1
AP2A2
AP2B1
AP2M1
AP2S1
ARF1
CANX
CAPZA1
CAPZA2
CAPZA3
CAPZB
CD74
CENPE
CLTA
CLTC
CTSD
CTSE
CTSF
CTSL
CTSS
CTSV
DCTN1
DCTN2
DCTN3
DCTN4
DCTN5
DCTN6

DNM2
DYNC1H1
DYNC1I1
DYNC1I2
DYNC1LI1
DYNC1LI2
DYNLL1
DYNLL2
FCER1G
FCGR2B
HLA-DMA
HLA-DMB
HLA-DOA
HLA-DOB
HLA-DPA1
HLA-DPB1
HLA-DQA1
HLA-DQA2
HLA-DQB1
HLA-DQB2
HLA-DRA
HLA-DRB1
HLA-DRB3
HLA-DRB4
HLA-DRB5
IFI30
KIF11
KIF15
KIF18A
KIF22
KIF26A
KIF2A
KIF2B
KIF2C
KIF3A
KIF3B
KIF3C
KIF4A
KIF4B
KIF5A
KIFAP3
KLC1
KLC2
LAG3
LGMN

MARCH1
OSBPL1A
PYCARD
RAB7A
RACGAP1
RILP
SAR1B
SEC13
SEC23A
SEC24A
SEC24B
SEC24C
SEC24D
SEC31A
SH3GL2
SPTBN2
THBS1
TRAF6
TREM2

Table S2. Genes for measuring adhesion score.

Genes: GO_LEUKOCYTE_ADHESION_TO_VASCULAR_ENDOTHELIAL_CELL
ADD2
CCL21
CCL25
CCL28
CCR2
CX3CR1
CXCL12
ELANE
ETS1
GCNT1
GOLPH3
ICAM1
IRAK1
ITGA4
ITGB1
ITGB7
KLF4
LEP
MADCAM1
MIR141
MIR146A
MIR155
MIR21
MIR221
MIR222
MIR31
MIR92A1
MIR92A2
MIRLET7G
NFAT5
PODXL2
PTAFR
RELA
RHOA
ROCK1
SELE
SELL
SELP
SELPLG
SPN
TNF

TRAF6
VCAM1

Table S3. Clinical information of skin donors for scRNAseq.

Donor	Age	Gender	Tissue site	Surgery	Batch
Healthy donor 1	18	male	limbs (forearm)	nevus removal	batch 1
Healthy donor 2	25	female	limbs (upper arm)	nevus removal	batch 1
Healthy donor 3	20	female	limbs (upper arm)	nevus removal	batch 1
Healthy donor 4	32	male	limbs (forearm)	nevus removal	batch 1
Healthy donor 5	38	male	limbs (upper arm)	skin flap transplantation	batch 1
Healthy donor 6	25	female	trunk (back)	nevus removal	batch 2
Healthy donor 7	45	female	trunk (breast)	reduction mammoplasty	batch 2
Healthy donor 8	23	male	foreskin	circumcision	batch 3, 4
Healthy donor 9	27	male	foreskin	circumcision	batch 3, 4
Healthy donor 10	28	male	foreskin	circumcision	batch 3, 4

Table S4. Clinical information of skin donors for immunofluorescence.

Donor	Age	Gender	Tissue site	Surgery
Healthy donor 1	33	male	limbs (forearm)	nevus removal
Healthy donor 2	24	female	limbs (thigh)	plastic surgery
Healthy donor 3	30	female	trunk (breast)	reduction mammoplasty
Healthy donor 4	31	male	trunk (abdominal)	skin flap transplantation
Healthy donor 5	43	female	trunk (breast)	reduction mammoplasty
Healthy donor 6	37	female	limbs (upper arm)	skin flap transplantation
Healthy donor 7	25	femal	trunk (back)	nevus removal
Healthy donor 8	29	male	limbs (upper arm)	skin flap transplantation
Healthy donor 9	20	male	foreskin	circumcision
Healthy donor 10	22	male	foreskin	circumcision
Healthy donor 11	27	male	foreskin	circumcision
